# Predictive Factors for the Healing of Rotator Cuff Repairs

**DOI:** 10.7759/cureus.67608

**Published:** 2024-08-23

**Authors:** Rodrigo A Beraldo, Jorge H Assunção, Paulo V Helito, Irline C Macedo Pontes, Mauro Emilio C Gracitelli, Caio Checchia, Fernando Brandão, Arnaldo A Ferreira Neto, Thaís Vasques, Eduardo A Malavolta

**Affiliations:** 1 Orthopaedics and Traumatology, Hospital das Clínicas da Faculdade de Medicina da Universidade de São Paulo (HCFMUSP), São Paulo, BRA; 2 Orthopaedics and Traumatology, Instituto Jundiaiense de Ortopedia e Traumatologia, Jundiaí, BRA; 3 Orthopaedics and Traumatology, Diagnósticos da América (Dasa) Hospital 9 de Julho, São Paulo, BRA; 4 Radiology, Hospital das Clínicas da Faculdade de Medicina da Universidade de São Paulo (HCFMUSP), São Paulo, BRA; 5 Radiology, Aspetar, Doha, QAT; 6 Orthopaedics and Traumatology, Hospital Sírio-Libanês, São Paulo, BRA; 7 Orthopaedics and Traumatology, Hospital do Coração (HCor), São Paulo, BRA

**Keywords:** preoperative factors, tendon integrity, predictive retear factors, combination of risk factors, arthroscopic rotator cuff repair, rotator cuff retear

## Abstract

Introduction: Rotator cuff tears (RCTs) are a significant cause of shoulder pain. Rotator cuff repair is common, but healing failure is frequent and the reasons for the retear are still unclear.

Objective: This study aimed to determine if specific preoperative factors related to patients and tears affect the structural outcome after rotator cuff repair.

Methods: This is a multivariate prognostic model study, based on prospectively collected data from a retrospective cohort. We included individuals who received arthroscopic RCT repair from January 2013 to April 2022. Evaluations were performed using magnetic resonance imaging (MRI) at 12 months postoperatively, and clinical outcomes were measured using the American Shoulder and Elbow Surgeons (ASES) scale. Statistical analysis was conducted using multivariate logistic regression.

Results: The sample included 176 patients, with a retear rate of 35.2%. Male (p=0.029), smoking (p=0.026), full-thickness infraspinatus tears (p=0.007), and instability of the long head of the biceps (p=0.046) were identified as predictive factors for non-healing. Traumatic lesions (p=0.017) favored healing. All patients showed significant clinical improvement. At 24 months, patients with healed tendons had better clinical outcomes.

Conclusion: Male sex, smoking, full-thickness infraspinatus tears, and instability of the long head of the biceps are predictive factors for retear after rotator cuff repair. Traumatic lesions favor tendon healing.

## Introduction

Shoulder pain has a high prevalence in the population, ranging from 7% to 26% [1]. Rotator cuff tears (RCTs), the main cause of shoulder pain, affect 20% of the general population and up to 50% of patients over 80 years old [2]. Rotator cuff repair is the primary reason for shoulder surgery [3]. However, healing failure after surgical repair remains an unresolved issue with retear rates ranging from 11% to 94% [4,5].

The analysis of predictive factors plays a crucial role in identifying patients with the potential for unsatisfactory outcomes after rotator cuff repair. Although there are some investigations into elements that contribute to an increased risk of non-healing [5-8] and adverse clinical outcomes [9-11], the literature is still scarce regarding the study of preoperative factors that may affect the prognosis of structural integrity. This includes the use of comprehensive samples and the application of multivariate logistic regression models for a more precise and predictive evaluation.

The objective of the study was to determine if specific preoperative factors related to the patient and the lesion would impact the structural outcome after rotator cuff repair.

## Materials and methods

Study design

We carried out a multivariate prognostic model study to assess healing following arthroscopic rotator cuff repair, utilizing prospectively gathered data from a retrospective cohort. The study was approved by Comitê de Ética em Pesquisa Plataforma Brasil (approval number: 73501123.3.0000.0068).

Study population

The study included patients who underwent arthroscopic RCT repair from January 2013 to April 2022. Surgical intervention was recommended for symptomatic patients with full-thickness tears unresponsive to conservative treatment, which included a minimum of three months of physiotherapy. Corticosteroid injections were not routinely administered, being reserved for patients experiencing severe pain.

The surgical procedures were conducted at a single hospital by four shoulder and elbow surgeons from the same institution, each with a minimum of seven years of experience performing these operations. The inclusion criteria encompassed primary arthroscopic rotator cuff repair, consistent collection of preoperative, perioperative, and postoperative data, as well as preoperative and postoperative magnetic resonance imaging (MRI) exams. The exclusion criteria involved partial rotator cuff repairs, repairs of partial tears, open or mini-open surgeries, and patients with prior surgeries on the same shoulder. Additionally, patients who did not undergo clinical evaluations at 12 and 24 months postoperatively were excluded.

Surgery and rehabilitation

The surgeries were performed in either the beach chair or lateral decubitus position, based on the surgeon's preference, utilizing general anesthesia and a brachial plexus block. Acromioplasty was conducted at the discretion of the surgeon. Treatment of the long head of the biceps was indicated in cases of instability (subluxation or dislocation) or partial tears greater than 25%. Tenotomy was performed in patients over 60 years old, while additional tenodesis was performed in younger patients, athletes, or individuals with a body mass index (BMI) less than 25, irrespective of age.

All tears were repaired using a single-row technique. Patients were immobilized with a sling for 4-6 weeks. Starting from the first postoperative day, movements of the elbow, wrist, and fingers were permitted. Passive exercises commenced after the third week, and active exercises began once the sling was removed. Muscle strengthening exercises were initiated after a significant improvement in the range of motion, typically around the 12th week. Patients were allowed to resume sports activities after six months, contingent upon the full restoration of range of motion and strength.

MRI

Preoperative and 12-month postoperative MRI data were collected by a musculoskeletal radiologist with five years of experience. Exams were performed using 1.5 or 3.0 Tesla magnets.

Outcomes

The primary outcome was tendon integrity at 12 months after surgery, according to the classification of Sugaya et al. [12]: Tendons were classified as "healed" (Sugaya type I, II, or III) and "not healed" (Sugaya type IV or V). Secondary outcomes were the scores of the American Shoulder and Elbow Surgeons (ASES) Standardized Shoulder Assessment Form [13] at six, 12, and 24 months postoperatively. 

Prognostic variables

We assessed the impact of 19 prognostic factors associated with patient and lesion characteristics, using tendon healing (classified as Sugaya type I, II, or III) as the dependent variable.

Patient-related factors

Patient baseline characteristics were age, sex, affected dominant side, diabetes, smoking, prior shoulder trauma, previous shoulder infiltration, and preoperative ASES score. Data were obtained through patient interviews conducted by the same research assistant one week before surgery.

Lesion-related factors

Lesion-related variables were assessed through MRI during the preoperative consultation. Supraspinatus tears were evaluated based on retraction in the coronal plane (<3 cm or ≥3 cm), anteroposterior extension (partial or full tendon involvement), and tears in the anterior portion. Infraspinatus tears were assessed by tendon thickness (intact, partial tear, or full-thickness tear), retraction (<3 cm or ≥3 cm), and anteroposterior extension (involving either the superior portion or the full tendon). The subscapularis tendon was categorized simply as "torn" or "not torn." Fatty degeneration in the supraspinatus, infraspinatus, and subscapularis muscles was graded from 1 to 3 according to Fuchs et al. [14].

The long head of the biceps was evaluated for rupture (none, partial, or complete) and instability (stable, subluxated, or dislocated, depending on its position within the bicipital groove, or not applicable in cases of complete rupture).

Missing data

No imputation techniques were employed for the variables studied as prognostic factors. For the ASES outcome, the last observation carried forward strategy was utilized. Patients who lacked results at 24 months, despite having reached the two-year mark post-procedure, had their 12-month data carried forward as the final result. When the 24-month result was available but preoperative, six-month, or 12-month results were missing, these were imputed using the mean. Patients without recorded scores at both 12 and 24 months were excluded from the analysis.

Sample size

Sampling was by convenience. According to the "Transparent Reporting of a Multivariable Prediction Model for Individual Prognosis Or Diagnosis (TRIPOD) [15]" guideline for writing and presenting data in prognostic studies, sample size calculation is not essential for this study design.

Statistical analysis

The association between prognostic factors and the Sugaya et al. classification was analyzed using logistic regression. After logistic regression, the model was adjusted using binomial regression to derive the effect size of each risk factor in the form of adjusted relative risk (RR). For each factor, one category was arbitrarily defined as the reference category (RR=1). For multinomial regression, an additional category of the dependent variable was used as the statistical reference, with the effect size presented as relative risk ratio (RRR).

The general characteristics of the sample were presented as mean and standard deviation for continuous data and as total value and percentage for categorical data. A p-value of less than 5% was considered significant. IBM SPSS Statistics for Windows, Version 20.0 (Released 2011; IBM Corp., Armonk, New York, United States) was used for the calculations.

## Results

**Figure 1:**
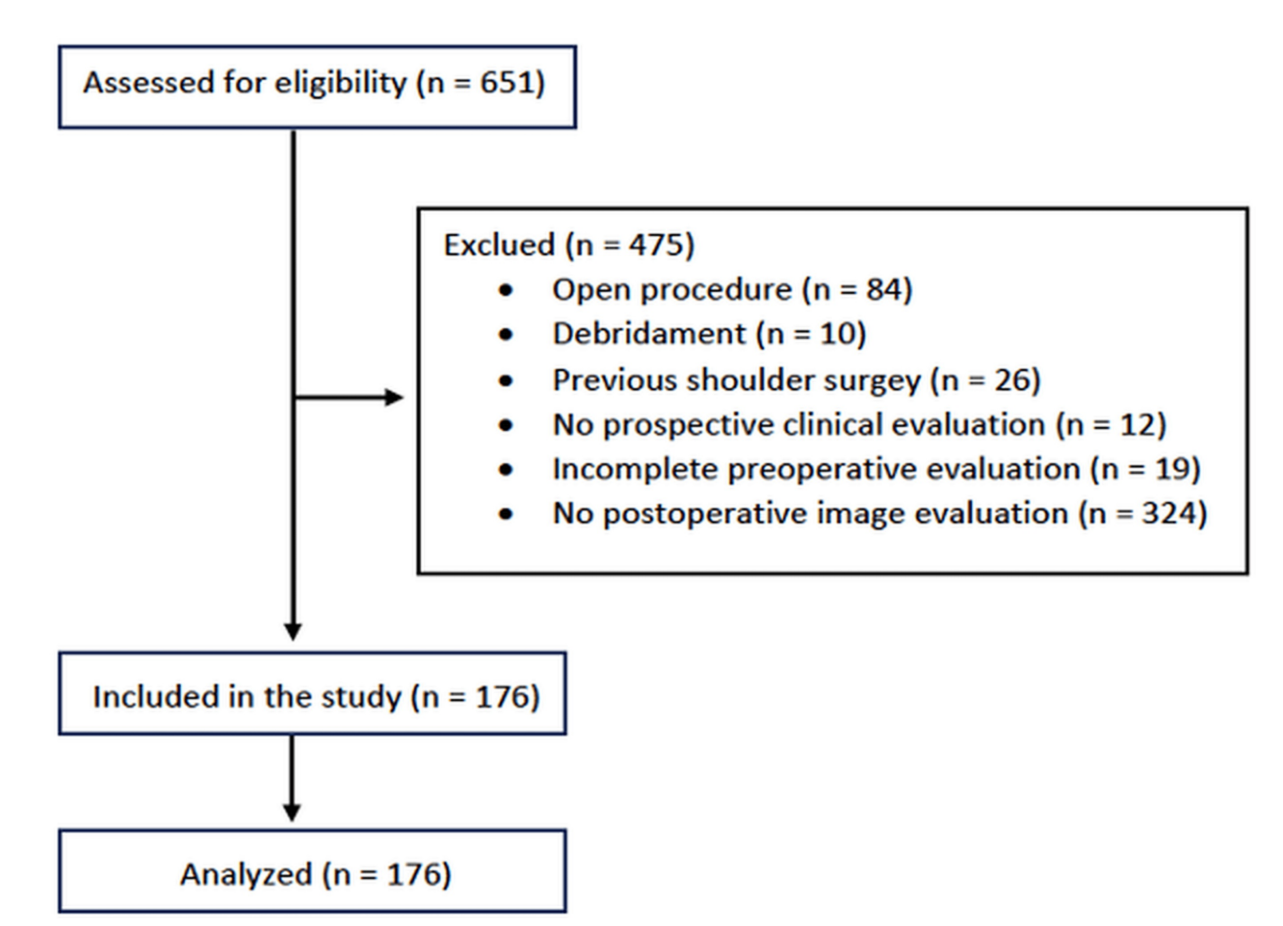
Flowchart of participant enrollment

During the evaluation period, 651 rotator cuff repair surgeries were performed. The exclusions were as follows: 84 open procedures, 10 debridement procedures, 26 cases with previous shoulder surgery, 12 patients without postoperative clinical evaluation, and 19 patients with incomplete preoperative evaluation data. Out of the remaining 500 patients, 324 were excluded due to the absence of MRI analysis of healing. Consequently, the analyzed sample comprised 176 patients (Figure 1). Data imputation was required for functional evaluation in four cases (2.3%) at six months, six cases (3.4%) at 12 months, and 11 cases (6.2%) at 24 months.

Patient and lesion characteristics

The retear rate found in our study was 35.2%. Of the 62 non-healed repairs, half were classified as Sugaya IV and half as Sugaya V (Table 1). The sample predominantly consisted of female patients, with an average age of 55.7 years, and most of the lesions involved the dominant side (Table 2). 

**Table 1:** Distribution by Sugaya classification

Sugaya	N (%)
I	7 (3.97)
II	34 (19.31)
III	73 (41.47)
IV	31 (17.61)
V	31 (17.61)

**Table 2:** Patient-related variables

	Healed (n=114)	Retear (n=62)	Total (n=176)	P-value
Age				
Mean±SD	54.6±8.2	57.7±8.2	55.7±8.3	0.017*
Sex				
Female	76 (66.7%)	32 (51.6%)	108 (61.4%)	0.054
Male	38 (33.3%)	30 (48.4%)	68 (38.6%)	
Side				
Right	83 (72.8%)	40 (64.5%)	123 (69.9%)	0.303
Left	31 (27.2%)	22 (35.5%)	53 (30.1%)	
Dominant side				
No	32 (28.1%)	24 (38.7%)	56 (31.8%)	0.176
Yes	82 (71.9%)	38 (61.3%)	120 (68.2%)	
Diabetes				
No	103 (90.4%)	51 (82.3%)	154 (87.5%)	0.153
Yes	11 (9.6%)	11 (17.7%)	22 (12.5%)	
Smoking				
No	83 (72.8%)	40 (64.5%)	123 (69.9%)	0.537
Ex-smoker	17 (14.9%)	12 (19.4%)	29 (16.5%)	
Smoker	14 (12.3%)	10 (16.1%)	24 (13.6%)	
Trauma				
No	102 (89.5%)	53 (85.5%)	155 (88.1%)	0.47
Yes	12 (10.5%)	9 (14.5%)	21 (11.9%)	
Infiltration				
No	93 (81.6%)	50 (80.6%)	143 (81.2%)	1
Yes	21 (18.4%)	12 (19.4%)	33 (18.8%)	

The supraspinatus tendon was involved in all cases, with full-thickness tears in 92.6% of cases and retraction >3 cm in 30.1% of shoulders (Table 3). Transfixing infraspinatus tears occurred in 16.5% of cases and subscapularis tears in 38.6%. Fatty infiltrations were mostly classified as Fuchs grade 1 (Table 4). The majority of patients (81.1%) had an intact long head of the biceps tendon (Table 4).

**Table 3:** Variables related to the supraspinatus

	Healed (n=114)	Retear (n=62)	Total (n=176)	P-value
Supraspinatus thickness				
Intact	0 (0%)	0 (0%)	0 (0%)	-
Partial articular	8 (7%)	0 (0%)	8 (4.5%)	
Partial intrasubstantial	1 (0.9%)	0 (0%)	1 (0.6%)	
Partial bursal	4 (3.5%)	0 (0%)	4 (2.3%)	
Full-thickness	101 (88.6%)	62 (100%)	163 (92.6%)	
Supraspinatus full-thickness				
No	13 (11.4%)	0 (0%)	13 (7.4%)	-
Yes	101 (88.6%)	62 (100%)	163 (92.6%)	
Supraspinatus retraction				
Small/medium	92 (80.7%)	31 (50%)	123 (69.9%)	<0.001
Large/extensive	22 (19.3%)	31 (50%)	53 (30.1%)	
Supraspinatus complete				
No	84 (73.7%)	33 (53.2%)	117 (66.5%)	0.008
Yes	30 (26.3%)	29 (46.8%)	59 (33.5%)	
Supraspinatus anterior part				
No	28 (24.6%)	9 (14.5%)	37 (21%)	0.127
Yes	86 (75.4%)	53 (85.5%)	139 (79%)	
Supraspinatus Fuchs				
1	92 (80.7%)	39 (62.9%)	131 (74.4%)	0.002
2	18 (15.8%)	11 (17.7%)	29 (16.5%)	
3	4 (3.5%)	12 (19.4%)	16 (9.1%)	

**Table 4:** Variables related to the infraspinatus, subscapularis, and long head of the biceps tendons

	Healed (n=114)	Retear (n=62)	Total (n=176)	P-value
Infraspinatus thickness				
Intact	92 (80.7%)	40 (64.5%)	132 (75%)	<0.001
Partial	13 (11.4%)	2 (3.2%)	15 (8.5%)	
Full	9 (7.9%)	20 (32.3%)	29 (16.5%)	
Infraspinatus retraction				
Small/medium	109 (95.6%)	53 (85.5%)	162 (92%)	0.037
Large/extensive	5 (4.4%)	9 (14.5%)	14 (8%)	
Infraspinatus extension				
Normal	92 (80.7%)	40 (64.5%)	132 (75%)	0.041
Upper portion	20 (17.5%)	21 (33.9%)	41 (23.3%)	
Complete	2 (1.8%)	1 (1.6%)	3 (1.7%)	
Fuchs infraspinatus				
1	103 (90.4%)	44 (71%)	147 (83.5%)	0.003
2	8 (7%)	11 (17.7%)	19 (10.8%)	
3	3 (2.6%)	7 (11.3%)	10 (5.7%)	
Subscapularis injury				
No	78 (68.4%)	30 (48.4%)	108 (61.4%)	0.01
Yes	36 (31.6%)	32 (51.6%)	68 (38.6%)	
Subscapularis Fuchs				
1	106 (93%)	51 (82.3%)	157 (89.2%)	0.043
2	6 (5.3%)	10 (16.1%)	16 (9.1%)	
3	2 (1.8%)	1 (1.6%)	3 (1.7%)	
Biceps injury				
Normal	102 (89.5%)	40 (65.6%)	142 (81.1%)	<0.001
Partial injury	10 (8.8%)	18 (29.5%)	28 (16%)	
Auto-tenotomized	2 (1.8%)	3 (4.9%)	5 (2.9%)	
Biceps stability				
Topic	99 (86.8%)	37 (59.7%)	136 (77.3%)	<0.001
Subluxated	13 (11.4%)	13 (21%)	26 (14.8%)	
Luxated	0 (0%)	9 (14.5%)	9 (5.1%)	
Auto-tenotomized	2 (1.8%)	3 (4.8%)	5 (2.8%)	

ASES scale

All patients showed a statistically significant clinical improvement postoperatively. There was no difference between patients with healed and non-healed tendons at the six- and 12-month evaluations (p=0.100; p=0.638). However, patients with healed tendons had a statistically superior clinical outcome at the 24-month evaluation (p=0.007), with an average score of 77.82 compared to 68.82 in the non-healed group (Table 5). 

**Table 5:** Comparison of the ASES scale between groups in the studied period ASES: American Shoulder and Elbow Surgeons

	ASES mean (95% CI)		Difference mean (95% CI)	
Time	Healed	Retear		P-value
Preoperation	37.71 (33.85; 41.58)	34.81 (29.58; 40.05)	2.90 (-3.61; 9.41)	0.381
6 months	70.83 (66.96; 74.69)	65.37 (60.13; 70.61)	5.46 (-1.05; 11.97)	0.100
12 months	73.47 (69.61; 77.34)	75.03 (69.79; 80.27)	-1.55 (-8.06; 4.96)	0.638
24 months	77.82 (73.95; 81.68)	68.82 (63.58; 74.06)	9.00 (2.49; 15.51)	0.007

Prognostic factors: multivariate logistic regression analysis

The independent factors for tendon non-healing were male sex, smoking, full-thickness infraspinatus tears, and instability of the long head of the biceps tendon. Traumatic tears were identified as a good prognostic factor for healing (Table 6). 

**Table 6:** Multivariate analysis results

	Multivariate		
	Odds ratio (95% CI)	P-value	
Age (increase in five years)	1.04 (0.99; 1.1)	0.154	
Male	2.92 (1.12; 7.66)	0.029	*
Dominant side affected	0.46 (0.2; 1.09)	0.078	
Diabetes	2.67 (0.78; 9.14)	0.117	
Smoking	3.74 (1.17; 12.01)	0.026	*
Traumatic injury	0.14 (0.03; 0.71)	0.017	*
Prior infiltration	2.23 (0.78; 6.32)	0.133	
Supraspinatus: large/extensive retraction	2.07 (0.7; 6.08)	0.187	
Supraspinatus: full-thickness	1.05 (0.38; 2.93)	0.919	
Supraspinatus: anterior portion involved	1.74 (0.59; 5.14)	0.318	
Supraspinatus: grade 2 fatty degeneration	0.53 (0.16; 1.74)	0.296	
Supraspinatus: grade 3 fatty degeneration	4.73 (0.61; 36.64)	0.136	
Infraspinatus: full-thickness	8.98 (1.8; 44.91)	0.007	*
Infraspinatus: large/extensive retraction	0.21 (0.03; 1.59)	0.130	
Infraspinatus: grade 2 fatty degeneration	2.65 (0.65; 10.8)	0.174	
Infraspinatus: grade 3 fatty degeneration	3.18 (0.37; 27.72)	0.294	
Injured subscapularis	0.56 (0.2; 1.51)	0.251	
Subscapularis: grade 2 fatty degeneration	3.23 (0.54; 19.4)	0.201	
Subscapularis: grade 3 fatty degeneration	0.1 (0; 5.66)	0.263	
Biceps injury			
Partial injury/auto-tenotomized	3.04 (0.99; 9.3)	0.052	
Subluxated/dislocated biceps	3.24 (1.02; 10.28)	0.046	*

## Discussion

In this study, we explored the prognostic factors that affect tendon healing after arthroscopic rotator cuff repair. Results have shown a concerning 35.2% rate of retears. This rate is higher than the 26.6% reported by McElvany et al. [16] in their meta-analysis. This finding may be attributed to the fact that most of these exams were performed on symptomatic patients, while many asymptomatic patients chose not to undergo an MRI.

Our finding that male sex negatively impacts tendon healing contrasts with the current evidence in the literature, as no studies with similar findings have been identified until now [17,18]. Conversely, Fancher et al. [19], through a meta-analysis of 11 studies with a total of 2,860 cases, did not detect significant differences in tendon healing between sexes.

In our analysis, smoking was shown to be a risk factor for non-healing. This finding is consistent with the results of Fan et al. [20] in a meta-analysis of 73,817 cases, which evaluated clinical and structural outcomes after rotator cuff repair in smokers, demonstrating a higher retear rate, but no difference in clinical scores. However, other similar studies failed to demonstrate this relationship [17]. 

Full-thickness infraspinatus tendon tears were also identified as a risk factor for non-healing. We believe this can be explained by the fact that these are simply larger lesions, since all patients had supraspinatus tears. Despite this, our multivariate analysis did not find statistical significance regarding either the extent of tears or fatty degeneration. However, most systematic reviews indicate that extent, retraction, and fatty degeneration are important variables for tendon healing [17,21]. 

Preoperative instability of the long head of the biceps, including subluxation or dislocation, negatively impacted rotator cuff healing. Our finding is in line with the publication by Zhao et al. [17], which concluded from a meta-analysis that the need for a procedure on the long head of the biceps is a risk factor for the non-healing of rotator cuff repair. However, some clinical trials [7] and systematic reviews [22] do not show a relationship between biceps lesions and rotator cuff retear. We believe that patients with instability of the long head of the biceps also have more extensive rotator cuff lesions, which may impact their healing.

Traumatic etiology of RCTs was found to be a good prognostic factor for tendon healing. This is in line with the publication by Paul et al. [23], in a prospective cohort study, demonstrating a higher healing rate and also better functional outcomes in patients with traumatic tears. However, some studies are controversial on this topic. Guevara-Alvarez et al. [24] showed no difference in tendon healing when comparing over 600 patients, with approximately half having traumatic etiology and the other half, degenerative tears. Similarly, Baum et al. [25] demonstrated comparable clinical and structural outcomes in patients with or without trauma after analyzing 973 cases. We believe that traumatic tears have a better prognosis because they generally affect younger patients with less fatty degeneration.

All other variables analyzed in our study did not show a statistically significant influence on tendon healing after multivariate analysis. Variables such as diabetes and fatty degeneration are commonly cited as relevant for the structural outcome after rotator cuff repair [17,26]. These variables showed high odds ratios in our study but lacked statistical significance, possibly due to our sample size not being large enough. On the other hand, there is a great variability of findings in the literature regarding risk factors for rotator cuff retear. These may be related to significant differences in study designs, sample sizes, types of imaging exams, and statistical models used. Perhaps for this reason, Saccomanno et al. [22] concluded in their systematic review that "it was not possible to reach any definitive conclusions about the most relevant predictors of rotator cuff repair outcomes."

We did not observe significant differences in clinical outcomes between patients with and without tendon healing at six- and 12-month evaluations. However, at 24 months, patients with healed tendons had significantly better clinical outcomes. This finding is in line with Karasuyama et al. [27], who demonstrated, in a meta-analysis of 26 articles with 3,072 cases, the importance of structural tendon integrity for good long-term clinical outcomes. However, Holtedahl et al. [28] concluded, after a meta-analysis of 3,350 patients, that although clinical outcomes are statistically better in patients with healed tendons, these findings have little clinical importance in patients' perception. We believe that tendon healing directly influences the maintenance of good long-term functional outcomes.

This study has several important limitations. Firstly, it is based on a retrospective cohort, and although data were collected prospectively, inherent biases in this design cannot be ignored. Despite evaluating a range of preoperative factors, not all variables that could potentially influence tendon healing were considered. Additionally, the majority of patients in our sample had isolated supraspinatus tears with minimal retraction. Therefore, caution should be exercised when generalizing these results to massive tears involving multiple tendons. Our study did not include consecutive cases. Additionally, we have a non-random loss bias, with asymptomatic patients more commonly failing to undergo MRI than symptomatic ones. Although our sample size is considerable compared to similar studies [6,29], it is still considered a small number of patients. Finally, our MRI evaluations were performed by only one radiologist on a single occasion, precluding analysis of possible interobserver and intraobserver differences.

On the positive side, we highlight the use of tendon healing evaluation performed by MRI assessed by a specialist with experience in musculoskeletal radiology. Additionally, the standardization of surgical techniques reduces the risk of finding differences in tendon healing that are related to intraoperative variables.

The use of scores like the Rotator Cuff Healing Index (RoHI) [30] is a current trend in clinical practice, providing an objective and integrated evaluation of prognostic factors. Our study can contribute to the improvement of these scores by providing further evidence on the relevance of variables such as smoking and instability of the long head of the biceps, thus enhancing the ability to predict healing and guide therapeutic decisions regarding RCTs.

## Conclusions

The prognostic factors for non-healing after arthroscopic repair of RCTs were male sex, smoking, full-thickness infraspinatus tears, and instability of the long head of the biceps. Traumatic tears were found to be a predictive factor for tendon healing. However, more prospective and randomized clinical studies are needed to better understand the prognostic factors for rotator cuff healing. These future studies will provide more robust and detailed data, allowing for a better understanding of the factors influencing recovery and improving treatment strategies.
